# Catatonia and Cognitive Impairments: A Systematic Review

**DOI:** 10.3389/fpsyt.2022.877566

**Published:** 2022-06-30

**Authors:** Francesc Serrat, Maria Iglesias-Gónzalez, David Sanagustin, Mikel Etxandi, Joan de Pablo, Jorge Cuevas-Esteban

**Affiliations:** ^1^Servei de Psiquiatria, Hospital Universitari Germans Trias i Pujol, Badalona, Spain; ^2^Departament de Psiquiatria, Universitat Autònoma de Barcelona, Barcelona, Spain; ^3^Consorcio de Investigación Biomédica en Red de Epidemiología y Salud Pública (CIBERESP), Madrid, Spain; ^4^Institut de Recerca Germans Trias i Pujol, Badalona, Spain; ^5^Centro de Investigación Biomédica en Red de Salud Mental (CIBERSAM), Madrid, Spain

**Keywords:** catatonia, cognitive impairments, executive function, frontal lobe, review

## Abstract

**Background:**

Catatonia is an underdiagnosed and undertreated neuropsychiatric syndrome characterized by catalepsy, negativism, mutism, muscular rigidity, and mannerism, often accompanied by autonomic instability and fever. Although there is growing interest in studying cognitive impairments before and after catatonia, little is known about the cognitive features of the syndrome.

**Methods:**

This systematic review was registered at PROSPERO (CRD42022299091). Using a Preferred Reporting Items for Systematic Reviews and Meta-Analyses (PRISMA) approach, we searched PubMed, ScienceDirect, and PsycArticles using a combination of the terms “Catatonia” and “Cognitive impairment” and “Executive function” and “Frontal lobe” and “Parietal lobe.” Studies included original research articles enrolling patients with catatonic syndrome according to specified criteria. Fourteen studies were deemed relevant for inclusion. The abstraction form included age, assessment during acute episode, associated diagnosis, assessment procedure, and cognitive domains. Outcome measures were extracted.

**Results:**

Executive functions and visuospatial abilities proved to be the most investigated domains. A great heterogeneity has been observed in the assessment tools used among the 14 evaluated studies. Findings showed that catatonic patients had worse performance than healthy and non-catatonic psychiatric patients in frontal and parietal cortical functions.

**Conclusion:**

Because of the small number of studies in such heterogeneous areas and significant methodological limitations, the results should be regarded with caution. Future research assessing cognitive impairments on catatonic patients is needed.

**Systematic Review Registration:**

[https://www.crd.york.ac.uk/prospero/display_record.php?RecordID=299091], identifier [CRD42022299091].

## Introduction

Catatonia is an underdiagnosed and undertreated neuropsychiatric syndrome first described by Kahlbaum in 1874, who defined it as “a motor syndrome occurring in association with affective disorders, epilepsy, and tuberculosis” (1). It’s characterized by catalepsy, negativism, mutism, muscular rigidity, and mannerisms, often accompanied by autonomic instability and fever. Volitional disturbances led to the classification of catatonia as a subtype of schizophrenia for most of the 20th century. However, revisions in nosology have recognized a great prevalence in mood disorders, their overlap with delirium, and comorbidity with medical conditions (2), and has finally been separated from schizophrenia in the Diagnostic and Statistical Manual of Mental Disorders, Fifth Edition (DSM-5) (3). Although the identification of catatonia is not difficult, since its classification has been controversial, it is often missed, leading to the false notion that the syndrome is rare. A lack of comprehension of catatonia’s phenotypes could be a major reason in underdiagnosis (4). Many classic illustrations of catatonia simply show it as a hypokinetic state (5); unfortunately, such representations may limit knowledge of catatonia’s many phenotypes. Catatonia prevalence in clinical samples was 9.0% in a recent review of 74 studies from all continents that collected data from 1935 to 2017 (6).

Although various hypotheses have been postulated, including neurotransmitter-related, infective, genetic, immunologic, metabolic, and psychological aspects, the pathophysiology behind catatonia remains unknown. Gamma-aminobutyric acid (GABA) and glutamate signaling have been proposed as causative variables (7). Benzodiazepine and electroconvulsive therapy (ECT) are the treatment of choice for catatonia. Still, a significant number of patients do not respond well to benzodiazepines (8).

Cognitive impairments range in severity from “mild,” which may be observed by the patient or healthcare providers, to “severe,” such as dementia, which interferes with daily activities or prevents a patient from functioning independently. Neuropsychological testing, which compares performance across several cognitive areas against age and sex standardized mean scores, is frequently used to identify cognitive impairment. Cognitive syndromes, such as dementia, are diagnosed clinically and according to a recognized classification system.

While the criteria for catatonia in the DSM-5 do not include cognitive impairments, the syndrome involves an underlying cognitive dysfunction that can be difficult to appreciate in the presence of unusual behavior and abnormal movements (9). Nowadays, the DSM-5 criteria for diagnosis requires 3 or more of 12 clinical symptoms; however, consistent reference definitions are lacking and the structure of catatonia is still unclear. Catatonics, unlike Parkinson’s patients, are unaware of their movement disturbances, which are likely caused by cognitive changes in attentional–motor interactions (10). Understanding the underlying mechanisms that cause cognitive impairment among catatonia is crucial to improve knowledge of prognosis and clinical management.

The brain structures affected in catatonia is not fully understood; however, the majority of neuroimaging studies have pointed to dysfunction in the frontal and parietal cortices (11). Despite Taylor’s assertion (12), that catatonia is a frontal lobe syndrome due to its role in attention, emotion control, and motor regulation, there are few detailed outcome studies of catatonia in terms of cognitive impairments, and the catatonia/frontal lobe association remains unclear due to a lack of clarity about the clinical epidemiology of cognitive impairment among catatonia patients. The purpose of this review is to deliver empirical support to the hypothesis that patients with catatonic syndrome will present specific cognitive impairments in the form of executive dysfunction. A comprehensive synthesis of all published literature can provide useful information in this case. The aim of this study is to deliver an extensive review of all studies dealing with the relationship between catatonia and cognitive impairments.

## Methods

### Search Strategy

The Preferred Reporting Items for Systematic Reviews and Meta-Analyses (PRISMA) guidelines was followed in conducting this study. The study protocol was registered in PROSPERO (CRD42022299091). The search was conducted in **PubMed**, **ScienceDirect**, and **PsycArticles** database up to December 2021, according to the PRISMA guidelines (13), using search criteria based on cognitive domains and measures of impairments on catatonic patients. The search strategy included terms related to: “*Catatonia, Cognitive impairment, Executive Function, Frontal lobe, Parietal lobe*.” The detailed electronic search strategy is displayed in Table 1. The reference section of the retrieved articles were also examined, to identify any additional relevant studies. The identified articles were imported into Zotero 5. After acquiring all records, the results were validated, and the duplicates were removed. Researchers with expertise in the topic of interest suggested relevant studies.

**TABLE 1:** Search strategy.

PubMed	ScienceDirect	PsycArticles
(Catatonia) AND (Cognitive impairments) *n* = 37	(Catatonia) AND (Cognitive impairments) *n* = 724	(Catatonia) AND (Cognitive impairments) *n* = 56
(Catatonia) AND (Cognitive impairments) AND (Executive Functions) *n* = 3	(Catatonia) AND (Cognitive impairments) AND (Executive Functions) *n* = 148	(Catatonia) AND (Cognitive impairments) AND (Executive Functions) *n* = 488
(Catatonia) AND (Cognitive impairments) AND (Executive Functions) AND (Frontal lobe) *n* = 1	(Catatonia) AND (Cognitive impairments) AND (Executive Functions) AND (Frontal lobe) *n* = 94	(Catatonia) AND (Cognitive impairments) AND (Executive Functions) AND (Frontal lobe) *n* = 278
(Catatonia) AND (Cognitive impairments) AND (Executive Functions) AND (Frontal lobe) AND (Parietal lobe) *n* = 0	(Catatonia) AND (Cognitive impairments) AND (Executive Functions) AND (Frontal lobe) AND (Parietal lobe) *n* = 56	(Catatonia) AND (Cognitive impairments) AND (Executive Functions) AND (Frontal lobe) AND (Parietal lobe) *n* = 198

### Inclusion Criteria

**FIGURE 1:**
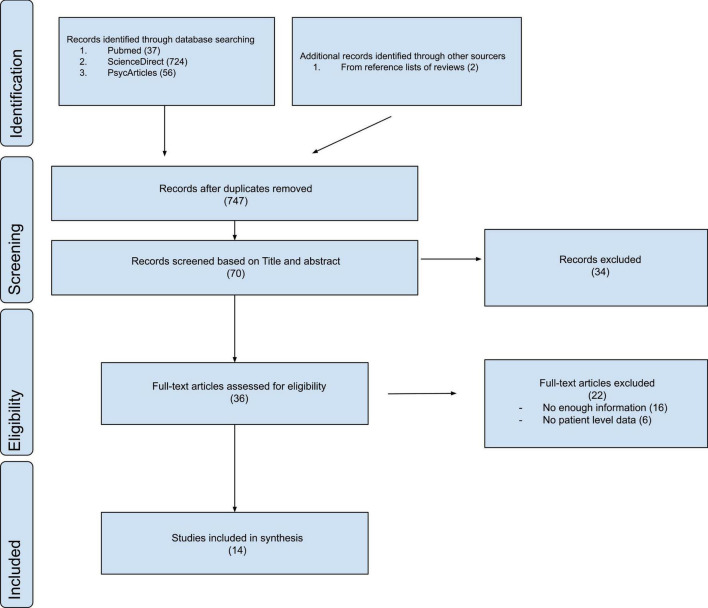
PRISMA flowchart.

We included all original work (articles, case reports, and studies) that reported studies dealing with the catatonia/cognitive impairments relation in humans with diagnosed catatonia. The studies examined in this review met the five criteria listed below: (1) original research articles that considered cognitive impairment in association with catatonia; (2) the study enrolled patients with catatonic syndrome; (3) the diagnosis was made according to specified criteria; (4) were published in English; and (5) were published in peer-reviewed journals. No other restrictions (e.g., date of publication) were applied. Review articles were excluded. The researchers screened in duplicate the articles identified in the search by first reviewing the tittle and abstract of the paper and, afterward, by reading the full-text paper. The detailed research strategy is presented in a flow chart Figure 1.

### Data Extraction

Studies that met the inclusion criteria were summarized in terms of: (1) participants (sample characteristics and sample size); (2) associated diagnostic; (3) specific cognitive domain assessed; (4) assessment procedure; (5) assessment during acute episode; and (6) results. The first author extracted data from the studies that were included, and an independent rater assessed for accuracy (JC-E). Articles were reexamined until consensus (100% agreement) was established in cases where the extracted data disagreed.

### Risk of Bias

Following “the Cochrane Collaboration’s tool for assessing risk of bias” (14), the probability of bias was reduced in the extraction of data and in ratings of study quality for this review. With this tool, we addressed the six collected domains with their corresponding specific items. The analysis of the risk of bias for each of the selected studies are summarized in Table 2.

**TABLE 2:** Risk of bias analysis.

	Selection bias	Selection bias	Performance bias	Detection bias	Attrition bias	Reporting bias
	
	Random sequence generation	Allocation concealment	Blinding (participants and personnel)	Blinding (outcome assessment)	Incomplete outcome data	Selective reporting
Starkstein et al. 18	+	?	+	+	+	?
Northoff et al. 19	+	+	+	+	?	+
Northoff et al. 17	+	+	+	+	+	+
Northoff et al. 16	+	+	+	+	+	+
Baker et al. 20	?	+	+	+	+	+
Bark et al. 21	+	+	+	+	+	+
Richter et al. 15	?	+	+	+	+	+
Utumi et al. 22	?	+	+	+	?	+
Kontaxaki et al. 23	+	−	+	?	?	−
Colomer et al. 24	+	+	+	+	+	+
Medina and Cooper 25	?	+	+	+	?	+
Graziane et al. 26	?	+	+	+	?	+
Dean et al. 27	+	+	+	+	+	+
Jiang et al. 28	?	+	+	+	+	+

*“+” low risk of bias; “−” high risk of bias; and “?” unclear risk of bias.*

## Results

### Database

Our search strategy revealed 817 potentially relevant manuscripts in PUBMED, SCIENCEDIRECT, and PSYCARTICLES. During the title and abstract screening, 747 were eliminated, leaving 70 full-text publications to be evaluated for eligibility. As a result of this phase, the present systematic review includes a total of 12 studies. Subsequent searches identified two supplementary studies. Figure 1 describes the evaluation of these 817 publications.

In total, 14 articles directly focusing on the relationship between catatonia and cognitive impairments met the inclusion criteria (see Table 3). The studies were ordered by date of publication and are categorized by the number of participants, age, assessment during acute episode, associated diagnosis, assessment procedure, cognitive domain, and results. The analysis of the risk of bias for each of the selected studies following “the Cochrane Collaboration’s tool for assessing risk of bias” (14), are summarized in Table 2.

**TABLE 3:** Features of the 14 studies reviewed.

Study	Number of participants (*n*)	Age	Assessment during acute episode	Associated diagnosis	Assessment procedure	Cognitive domains	Results
Starkstein et al. 18	*N* = 120 79 with depression 41 with Parkinson’s	51–64	No	Depression	M.M.S.E	Orientation Attention Visual construction	Frontal dysfunction induced by frontotemporal lobe atrophy may be linked to catatonia.
Northoff et al. 19	*N = 39* 13 catatonic 13 controls 13 non-catatonic psychiatric	43.4 ± 13.1 (C) 50.4 ± 15.3 (nC) 46.5 ± 9.2 (nCP)	No	None	Standard Progressive Matrices D2 Attention Test, Color-Word Interference Test Visual Object and Space Perception Test Observation from the Wilde Test Trail Making Test, Five Point Test, Verbal Fluency Test, Box Piling Test Progressive arithmetics.	Attention Executive functions Working memory Visual–spatial abilities	In visual–spatial ability, working memory and attentional-motor functions catatonic individuals demonstrated significantly lower performance and distinct correlation patterns.
Northoff et al. 17	*N* = 30 10 catatonic 10 controls 10 non-catatonic psychiatric	41.6 ± 5.3 (C) 40.1 ± 6.2 (nC) 40.8 ± 4.9 (nCP)	No	Schizophrenia Affective disorders	Standard Progressive Matrices D2 Attention Test, Color-Word Interference Test Visual Object and Space Perception Test Trail Making Test, Five Point Test.	Attention Visual–spatial abilities Executive functions	Only in visual–spatial abilities did catatonic patients vary from psychiatric and healthy controls, with considerably worse performance and abnormal intercorrelations with attentional measures.
Northoff et al. 16	*N* = 30 10 catatonic 10 non-catatonic 10 non-catatonic psychiatric	41.6 ± 5.3 (C) 40.8 ± 4.9 (nC) 25.9 ± 6.1 (nCP)	No	Akinetic catatonia	None (brain imaging).	Affective inhibition	During emotional processing, catatonic symptoms may be associated to dysfunction in the orbitofrontal cortex and subsequent changes in the prefrontal cortical network.
Baker et al. 20	*N* = 1	46	No	Depression	WAIS III Adult Memory and Information Processing Battery Camden Memory Test CANTAB Rey Complex Figure/Taylor Figure Trail Making Test Semantic Fluency Test.	Memory Attention Executive functions	The findings support existing definitions of catatonia as a frontal condition marked by persistent impairment of executive function, but there was also evidence of severe anterograde amnesia.
Bark et al. 21	*N* = 53 8 catatonic schizophrenia 19 paranoid schizophrenia 26 control	29.81 ± 9.39 (C) 36.75 ± 10.48 (CS) 38.5 ± 13.99 (PS)	No	Schizophrenia	The Iowa Gambling Task Wisconsin Card Sorting Test Object Alternation Task Go-NoGo task The Standard Progressive Matrices.	Attention Executive functions Working memory	According to preliminary findings, catatonic schizophrenia suffers from a unique deficiency in neuropsychological measures related to ventral prefrontal cortical function.
Richter et al. 15	*N* = 6	41.6 ± 5.3	No	None	None (brain imaging).	Affective inhibition	Lorazepam administration causes a modulation of the BOLD-response in the OFC during emotional processing. Lorazepam, in particular, was found to normalize signal reduction in catatonic patients as compared to healthy controls.
Utumi et al. 22	*N* = 3	63–74	No	Bipolar mood disorder	M.M.S.E	Mental rigidity Cognitive flexibility	Frontal dysfunction induced by frontotemporal lobe atrophy may be linked to catatonia.
Kontaxaki et al. 13	*N* = 91 71 schizophrenia 20 controls	30.23 ± 7.71	Unknown	Schizophrenia	Unknown	Response inhibition Declarative memory Executive functions	There was no link between hallucinations, highly organized delusions, persecutory delusions, agitation, catatonia, or inappropriate affect and any form of cognitive impairment.
Colomer et al. 24	*N* = 21	26.78	No	First-episode psychosis antipsychotic-naive	The MATRICS Consensus Cognitive Battery	Processing speed Attention Working memory Verbal learning Visual learning Reasoning	No differences in cognitive performance between catatonic and non-catatonic patients, although catatonic patients scored lower in all domains.
Medina and Cooper 25	*N* = 1	40	During acute state and after lorazepam challenge	Bipolar mood disorder	Clock Drawing Test	Visual–spatial abilities Executive functions	Greater catatonic severity is associated with greater cognitive dysfunction.
Graziane et al. 26	*N* = 1	66	During Acute state and after administration of memantine	Bipolar mood disorder	Montreal Cognitive Assessment	Executive and visuospatial functioning Language Attention Memory Orientation	Memantine improves both catatonic symptoms and co-occurring cognitive impairment.
Dean et al. 27	*N* = 172 43 catatonic 43 non-catatonic schizophrenia 86 control	27.4 ± 8.15 (C) 27.9 ± 6.63 (nC) 29.9 ± (nCP)	No	Schizophrenia	Screen for Cognitive Impairment in Psychiatry	Verbal Fluency Processing speed Memory	When compared to patients without a history of catatonia, people with a history of catatonia exhibit more cognitive difficulties with verbal fluency and processing speed.
Jiang et al. 28	*N* = 1	74	During acute state and after administration of clonazepam	None	Unknown	Executive function Cognitive control	After 5 months on clonazepam, testing revealed that the executive dysfunction had nearly resolved, with very minor residual deficits.

*C, catatonic; nC, non-catatonic; nCP, non-catatonic psychiatric; CS, catatonic schizophrenia; PS, paranoid schizophrenia.*

### Study Characteristics

The details of the included studies are shown in Table 3. Overall, all studies (*n* = 14) included in this review aimed to identify factors associated with cognitive impairment and catatonia in order to understand the impact on patients outcome. There was wide variation across all studies regarding cohort sizes (the sample size ranged from 1 to 172) and neuropsychological and/or cognitive assessment in terms of measures or tools used (see Table 4). Four studies (28.57%) were from the United States, eight (57.14%) from Europe, one (7.14%) from South America, and one (7.14%) from Asia. The mean age across the 14 reviewed studies was 45.5 ranging from 26.8 to 67.6 years. Only four studies (28.57%) included ages over 60 years old. Two studies used neuroimaging (15, 16), one used a combination of neuroimaging and neuropsychological assessment (17), while the rest used cognitive assessment tools (18–28). Only three studies conducted assessments during the initial acute period and the chronic period (25, 26, 28), whereas the rest of the studies reported conducting the assessment during the chronic period. There was one study that did not report the timing of when assessments were conducted (23). Affective disorders (35.71%) and psychotic disorders (35.71%) were the most prevalent underlying conditions, while two studies did not specify the underlying disease (19, 28). No study present in this review has taken into account the somatic comorbidity.

**TABLE 4:** Neuropsychological tests used across cognitive domains.

Cognitive domain	Measure
General intellectual functioning	1. Standard Progressive Matrices (SPM) 2. Multiple Vocabulary Test-B (MWT-B)
Visual–spatial abilities	1. Observation from the Wilde Test (BO-WIT) 2. Visual Object and Space Perception Test (VSOP) 3. Subtest 7 and 9 from the performance investigation systems (LPS) 4. Clock Drawing Test
Executive functions (general)	1. Rey Complex Figures Test-copy trial 2. Clock Drawing Test 3. Five Point Test (5PT)
Executive functions (attention)	1. D2 Attention Test 2. Color-Word Interference Test (CWI) 3. Trail Making Test A and B (TMT) 4. WAIS digit span 5. WAIS digit symbol 6. WAIS spatial span forward
Executive functions (working memory)	1. The MATRICS Consensus Cognitive Battery 2. Progressive arithmetics 3. WAIS digit span 4. WAIS spatial span 5. WAIS spatial working memory task 6. Object Alternation Task (OAT)
Executive functions (processing speed)	1. Trail Making Test A
Executive functions (decision making)	1. The Iowa Gambling Task
Executive functions (planning)	1. Box-Piling Test
Executive functions (response inhibition)	1. Go-NoGo task
Executive functions (cognitive flexibility)	1. Trail Making Test B 2. Wisconsin Card Sorting Test (WCST) 3. Two-group color test
Memory	1. Adult Memory and Information Processing Battery 2. Rey–Osterrieth Complex Figure Test 3. WAIS digit span 4. WAIS picture naming 5. WAIS similarities 6. Camden Memory Test
Overall cognitive assessment	1. M.M.S.E 2. MoCA 3. Screen for Cognitive Impairment in Psychiatry (SCIP) 4. CANTAB

*WAIS, Wechsler Adult Intelligence Scale; MoCA, Montreal Cognitive Assessment; CANTAB, Cambridge Neuropsychological Test Automated Battery; MMSE, Mini-Mental State Examination.*

### Neuropsychological Studies

Table 4 shows the various aspects of cognition that were examined across the studies. General intellectual functioning, visual–spatial abilities and executive functions (EFs) were frequently measured across the studies. Neuropsychological test measures were frequently used to identify subjectively reported cognition. The only neuropsychological instruments conducted in more than one study were the Trail Making Test (TMT) (17, 19, 20), the d2 Attention Test (17, 19), the Color-Word Interference Test (17, 19), and the Five Point Test (17, 19). In terms of EF, 22 different measures or sub-tests were used. Measures infrequently capture overall cognition function, while some test measures were used to assess as many as three cognitive domains. For example, the Wechsler Adult Intelligence Scale (WAIS) digit span was used to assess attention, working memory and memory.

On seven studies assessments were conducted using global cognition measures. Starkstein et al. (18) and Utumi et al. (22) studies, used the Mini-Mental State Examination (MMSE) to evaluate the cognitive state of their subjects. Both reports revealed poorer performances on catatonic patients, specially regarding attention and visual construction domains. The Starkstein study compared catatonic patients with mood disorders with non-catatonic Parkinson’s patients, while Utumis report only assessmented on three patients without control group. Medina et al. (25), applied the Clock Drawing Test on one catatonic patient, the assessment were conducted during the catatonic acute state and after deliver of lorazepam, revealing severe impairments on executive planning and visuospatial construction during the acute state. A similar study conducted by Graziane et al. (26), using the Montreal Cognitive Assessment tool (MoCA) on a single patient during the catatonic acute state and after delivering of memantine, showed similar results; cognitive dysfunction improving after treatment. Another similar study, assessing cognition during the acute state and after 5 months on clonazepam on a single patient, was conducted by Jiang et al. (28). The assessment tool was undisclosed, but the results were similar to the ones discussed; executive dysfunctions during the acute state that improved after treatment administration. Using the Screen for Cognitive Impairment in Psychiatry (SCIP) test, Dean et al. (27), compared schizophrenia patients with a history of catatonia to schizophrenic patients without a history of catatonia and a healthy control group. While both schizophrenic groups were shown to be impaired in all cognitive areas when compared to healthy control participants, the catatonic group performed considerably worse on verbal fluency and processing speed assessments. Colomer et al. (24), assessed six catatonic patients using the MATRICS Consensus Cognitive Battery (MCCB) and confronted them with fifteen non-catatonic patients (both groups suffered first-episode non-affective psychosis), their results did not find differences in cognitive performance between catatonic and non-catatonic patients, although catatonic patients scored lower in all domains.

The other five studies included in this review used tailored neuropsychological assessment tests, being the visuospatial abilities and the EF the main cognitive domains assessed. Northoff et al. (19) presented a study, where catatonic patients were compared to psychiatric non-catatonic patients (matched with regard to underlying psychiatric diagnosis; schizophrenic or affective psychosis) and healthy controls. The researchers employed a battery to evaluate a variety of neuropsychological abilities that are presumed to depend on frontal (EFs, attention, working memory) and parietal (visuospatial abilities) cortical function. Their findings demonstrated that catatonic patients varied solely in visuospatial ability from psychiatric and healthy controls, with considerably worse performance and intercorrelations with attentional measures. Baker et al. (20), reported a detailed neuropsychological evaluation in a single case of catatonia almost 3 years after recovery from the acute episode. A battery of tests, assessing general intellectual abilities, memory, EF, and visuospatial abilities, was performed 2 weeks after patients last ECT and repeated 2 years and 8 months later. Selective deficits in EF and anterograde amnesia were evident on the first evaluation and continued to be present at follow-up after almost 3 years, and cannot be explained convincingly by the administration of ECT due to the relatively small number of sessions received. Patients with catatonic schizophrenia were compared to patients with paranoid schizophrenia and healthy volunteers by Bark et al. (21). Their team performed a neuropsychological assessment that focused on EF presumed to be related to the function of the ventral prefrontal cortex. General intellectual functioning, visuospatial abilities, and attention were also assessed. Their results revealed specific deficits in decision making and set-shifting in catatonic patients in contrast to healthy and paranoid schizophrenic subjects. Finally, a study conducted by Kontaxaki et al. (23), run and undisclosed battery of neuropsychological tests in patients of the schizophrenic spectrum. Their results indicate that hallucinations, highly organized delusions, persecutory delusions, agitation, catatonia, and inappropriate affect did not associate with any subtype of cognitive deficit.

### Mixed Studies

Only one study investigated catatonia and cognitive impairments using a combination of neuroimaging and neuropsychological assessment. Northoff et al. (17), conducted a combined investigation, using a single photon emission computerized tomography (SPECT) and cognitive measures (d2 Attention Test, Visual Object and Space Perception Test, TMT, and Standard Progressive Matrices), on 10 catatonic patients, 10 psychiatric controls (with similar underlying psychiatric conditions but without catatonia), and 10 controls. This study revealed a significantly lower regional cerebral perfusion (r-CBF) in right lower prefrontal and parietal cortex in catatonia than in psychiatric and healthy controls, as well as poorer performance in visuospatial abilities associated with right parietal function.

### Neuroimaging Studies

The two neuroimaging studies of this review, were conducted using functional brain magnetic resonance imaging (fMRI) technique. On the study conducted by Northoff et al. (16), after successfully treating a catatonic episode with lorazepam, patients and controls in the study were required to complete an emotional regulation test. Changes in orbitofrontal cortex (OFC) activation were linked to abnormal functional connectivity between the OFC and the medial prefrontal cortex (mPCF), as well as between prefrontal and motor areas, according to the findings. The findings of Ritcher et al. (15) study, demonstrated that lorazepam might be used to treat abnormal activation of the OFC during an emotional task.

## Discussion

In this review, we aimed to identify studies that investigate the relationship between catatonia and cognitive impairments by analyzing the evidence from 14 studies meeting a criteria defined in the methods section. EF, attention and visuospatial abilities are among the most assessed cognitive domains, and the results lend evidence to support the frontal lobe syndrome theory proposed by Taylor (12), but it is worth mentioning that the studies revealed some limitations. First, we only found one study with a large sample size of *n* = 172 participants (27). All other studies reviewed used smaller samples ranged from *n* = 120 to *n* = 1. As a result, limited sample sizes may result in false negatives and/or restrict the capacity to identify cognitive deficits in some of the assessed domains. Moreover, it is not clear that some of the subjects in the Northoff et al. (17, 19) studies, were distinct in each study, resulting in the impression of overly robust results.

Furthermore, the majority of the studies included in this systematic review were conducted in Western countries, according to the findings. There were too few studies from non-Western countries (22), to make any meaningful comparisons. In order to tackle this geographical distribution bias, further cross-cultural research is needed to understand the EF deficits on catatonia within different cultural and geographical settings.

As age is an important factor for cognitive impairment, cognitive changes should be reviewed separately in young or adult. Surprisingly, we only found two studies with participants older than 65 (22, 26). There have been a few reports on the association between frontotemporal dementia (FTD) and catatonia (29, 30), since the diagnostic criteria for catatonia and FTD partly overlap (31). Catatonic symptoms are not unusual in FTD, according to Northoff et al., because catatonia is associated to frontal dysfunction (16), but this relationship is still not clear due to lack of studies.

This research reveals that a wide range of measures have been used to assess cognitive impairments in relation to different domains, particularly on EF, and the constructs studied are heterogeneous. It should be highlighted, however, that the current study does not examine whether those measures are valid or reliable in detecting cognitive deficits. For example, the study conducted by Northoff et al. (19), revealed no significant differences on EF impairments between catatonics and psychiatric controls while the results of Bark et al. (21), suggest a specific deficit in EF measures associated with decision making in catatonic schizophrenic patients when confronted with paranoid schizophrenia. A possible explanation for the controversial results is that open-ended tasks compared to more structured task may be more sensitive to reveal group differences in some cognitive domains like EF. Another relevant aspect, that can explain these results, is that while decision making may be related to dysfunction in the ventral prefrontal cortex, other EF domains, like attention, are more related with the dorsolateral prefrontal region (32).

The precise brain mechanisms that could be the source of the symptoms are very poorly known. Ritcher et al. (15) and Northoff et al. (16), used brain imaging to demonstrate that emotional regulation, functional connectivity, and the GABAergic system play a significant role in catatonic patients, but the exact neurophysiological and neuropsychological mechanisms of catatonia remain unclear, since there is a lack of combined studies of neuropsychology and neuroimage. We only found one study combining these factors (17), and while the results pointed the role of the right parietal cortex in catatonia, showing a significant decrease of r-CBF, and are at the same time supported by results from neuropsychological measures, the study suffers from methodological limitations, and more combined studies are needed to clarify this relationship.

While most of the studies focused their attention on working memory, other forms and processes of memory where only considered in studies using global cognition measures. Only one study conducted by Baker et al. (20), performed a detailed longitudinal neuropsychological assessment. As commented before, the results pointed to a permanent cognitive impairment of the patient, focally affecting memory and EF. Rather than a pure amnesic state, focal frontal lesions tend to result in a failure to use memory methods to improve coding and recall (33), but due to the small size of the study (*n* = 1), this assumption of memory impairments on catatonic patients due to EF dysfunction should be treated as a working hypothesis awaiting further empirical support.

To the best of our knowledge there are no studies dealing with the relation between somatic disorders and catatonia. Since catatonia can have a somatic etiology, it would be important to control for somatic comorbidity. None of the reviewed studies have taken into account the role of somatic factors in the etiology of catatonia and the cognitive implications that this may entail.

Leaving aside these discrepancies, the most promising cognitive domains impairments related to catatonia to explain Taylor’s frontal lobe syndrome theory were EF, attention and visuospatial abilities. Because of the small number of studies in such heterogeneously broad domains and methodological limitations, findings should be interpreted with caution. However, all those results linking EF with catatonia support its potential as an endophenotype.

Executive function has measurable behavioral effects (34), and is linked to genetic (35), and neurobiological mechanisms (36). Neuropsychological measures of EF have been associated to activation of brain areas such as the frontoparietal (35) and frontal cortical areas (37), according to functional imaging studies. The neural substrates of GABA and glutamate present a neural link for the EF (common factor), which has a genetic basis but may be assessed using cognitive tasks (38). In addition, genetic factors account for about half of the variation in EF performance (39). In summary, research on EF reveals that it fulfills the concept of endophenotype, and this could be a crucial piece in determining whether executive dysfunctions are a cause or a consequence of catatonia. We propose that a model of EF in catatonia that bridges the pathway from genetics to neural circuitry and to be observed EF phenotype may better capture the heterogeneity of EF in catatonia.

Conclusion is that the relationship between catatonia and cognitive impairments is still not fully clarified, and more advanced research may help develop better understanding of frontal lobe syndrome. To detect subtle subclinical deficits in the target population, future studies should utilize a more comprehensive and quantitative framework with more robust measures. Moreover, to gain an overview of all cognitive impairments related to catatonia, future studies should included samples across the whole age, and need to be sufficiently large to have enough power to detect group differences. This wide range of cognitive alterations found, as well as many others to be assessed, may underly multiple pathobiological mechanisms that should be taken care in future studies. Another important factor would be the need to control the somatic comorbidity and the importance of differentiating between acute, post-acute and chronic period. Functional neuroimaging studies can support neuropsychological information during acute periods due to the difficulty of obtaining information from patients when acute catatonia is present, specially in severe hypokinetic or hyperkinetic states. Regarding the control of the effects of benzodiazepines on neuropsychological examinations, it should be taken into account for future studies, what medication each subject was taking before and during hospital admission. It will also be interesting to monitor which medication is administered at the time of catatonia assessment and during cognitive examination. Studies on cognitive impairments on catatonia will continue to provide important insights by bringing researchers closer to the genetic etiology and neurobiological pathways underlying catatonia.

## Data Availability Statement

The datasets presented in this study can be found in online repositories. The names of the repository/repositories and accession number(s) can be found in the article/supplementary material.

## Author Contributions

FS developed the study concept, selected the manuscript, and reviewed the clinical data under the supervision of JC-E and MI-G. All authors reviewed and approved the final manuscript.

## Conflict of Interest

The authors declare that the research was conducted in the absence of any commercial or financial relationships that could be construed as a potential conflict of interest.

## Publisher’s Note

All claims expressed in this article are solely those of the authors and do not necessarily represent those of their affiliated organizations, or those of the publisher, the editors and the reviewers. Any product that may be evaluated in this article, or claim that may be made by its manufacturer, is not guaranteed or endorsed by the publisher.
